# Abstracts and Gleanings

**Published:** 1882-02-20

**Authors:** 


### ABSTRACTS AND GLEANINGS.

**Susceptibility to Variola.**—Dr. Porterfield, in Medical and Surgical Reporter, says: I find among the laity an almost universal opinion, and, indeed, among some physicians, that an attack of any of the zymotic diseases in the pregnant female will exempt the child from future attack. But from my own experience, and others which I have seen, I am led to the conclusion that if such is the rule, the exceptions to it are so numerous that but little dependence can be placed upon it as a prophylactic means. Of the considerable number of cases I have collected I will cite but three.

CASE 1.—Mrs. N., primipara, aged 27, was attacked with anginose variety of scarlatina, about the eighth month. About five days after the onset of the fever she was delivered of a pretty large boy, presenting a characteristic rash all over his chest, neck and face; this subsided and disappeared in about two days, the child showing no further symptoms, but died in its third year of the same disease in the malignant form.

CASE 2.—Mrs. R., during her second pregnancy, was attacked with the measles—this was during the fourth month. She went to her full term, the child showing no evidences of the malady, but it subsequently did have a well defined case of the same disease.

CASE 3.—Mrs. P. was vaccinated during her first pregnancy, and so violently did the virus attack her that she was confined to her bed for two weeks; the scars (two in number) are as large as a silver half dollar. The child was recently vaccinated by myself, and it worked very successfully, a well marked scar showing in each place.

Such cases as these, and others that have come to my notice, convince me that little dependence can be placed in the fact that the pregnant female has had a contiguous disease, or that she has been vaccinated while in this condition. I believe that in every person who has a contagious disease there is a special affinity for the poison of that particular disease, and that this affinity is an abnormal condition, a disease, so to speak, the conditions necessary to the existence of which we are at present in ignorance of, and hence we cannot say whether or not it may exist more than once. I believe that an attack of the disease exhausts this affinity and thus renders the subject exempt till the condition re-exists. Believing this, it is easy for me to see why many cases occur where the child fails to be exempt, even where the mother has been attacked while it was a fœtus in utero.

**Small-Pox Pustulation in the Fœtus in Utero.**—In reply to the question whether true pustulation of variola can occur in the fœtus in utero Dr. R. H. Day, of Baton Rouge, says in Maryland Medical Monthly: In the winter of 1831 and 1833, in the lower end of Calvert county, Maryland, in that portion adjacent to the Patuxent river, just below the mouth of St. Leonard's creek, upon the plantation of Dr. McGill, of Fredericksburg, Maryland, there occurred a number of cases of small-pox, some mild, others confluent and grave; among the latter was a negro woman, some 30 years of age, near the end of gestation.

She gave birth to her child some ten or twelve days after the eruptive stage of her disease, and perhaps two or three weeks of her full term.

I was with her at her confinement. The fœtus was evidently living up to within a short time of its birth, as the mother had felt its motions distinctly within a few hours of her delivery; and when born there was no appearance whatever to throw discredit upon the mother's statement; but my eyes looked upon a sickening spectacle—a fœtus covered with a full and distinct crop of small-pox pustules from head to foot, some of them already ruptured, and the excavations filled with pus, and many still unruptured, so characteristic as to mark the true nature of the eruption at first sight.

I very carefully examined the fœtus with enthusiastic interest, and desired mueh to preserve it as a pathological specimen, but in the country I had neither jar nor alcohol in which to keep it.

**The Bone-Conduction of Sound.**—In the New York Medical Journal and Obstetrical Review for February, 1882, Dr. J. A. Andrews, Assistant Surgeon to the Manhattan Eye and Ear Hospital, gives an account of his investigations in regard to the intermittent perception of sound, as conveyed through the cranial bones—the observations having been mostly clinical, largely with the use of the tuning-fork. In order that an explanation for the phenomenon of intermittent bone conduction may be understood, he thus formulates the points in differential diagnosis between an affection of the middle ear and one of the labyrinth, as evidenced by examination with the tuning-fork: 1. If a vibrating tuning-fork, c, be placed between the teeth, the hearing power being normal on one side and diminished on the other, and its tone be intensified in the ear of which the hearing power is diminished, the cause is seated in the external or middle ear, and the labyrinth is unaffected. 2. If the hearing power be impaired in both ears, and the sound of the tuning-fork be heard better in the worse ear, and intensified on closure of the ear of which the hearing power is most impaired, the cause is still located in the middle ear. 3. If under either of the above-mentioned conditions the vibrations of the tuning-fork be not heard better in that ear of which the hearing power is most impaired, even if its meatus be closed with the finger, and middle-ear disease as a cause can be excluded, there is an affection of the central apparatus of hearing. If the tone of the tuning-fork be still intensified by closure of the ear of which the hearing power is least impaired, there is disease of the eternal apparatus on one side only. Should the sound of the tuning-fork not be intensified by closure of either ear, then the disease is on both sides, and has its seat in the labyrinth or in the brain. In the first and second propositions the increased resonance results from the reflection of the vibration from the cranial bones upon the nerve. In the third proposition the reflection or condensation of the vibrations of the tuning-fork upon the nerve when the meatus is closed does not intensify their perception, because the function of the auditory nerve itself and not that of the conducting apparatus is impaired. The peculiarity that in some cases of middle-ear disease the watch is not heard by bone-conduction, and in other cases examination with the tuning-fork gives the signs of labyrinth disease—i. e., the tuning-fork being heard by bone-conduction better in the ear which is normal as to hearing power, therefore diminished instead of increased in the ear of which the hearing capacity is impaired—cannot, it seems to him, be explained by assuming an interference with the conduction through the chain of ossicles. He inclines to the belief, based upon experiments, that this phenomenon is due to increased intra-labyrinthine pressure, brought about in those cases of middle-ear disease in which there is an accumulation of fluid in the tympanum, or the membrana tympani is much depressed, in the former instance by the fluid in the cavity acting upon the oval or round window, or both, and in the latter instance by the plate of stapes being forced against the membrane in the oval window. In both cases the terminations of the acoustic nerve suffer a mechanical irritation which gives rise on the one hand to subjective noises in the ear, and on the other hand annuls the perception of certain tones. Extreme pressure upon these parts may so interfere with intra-labyrinthine vibrations as to completely obliterate bone-conduction from the tuning-fork.—[N. Y. Med. Journal and Ob. Review.

**Cold Applications in Typhoid Fever.**—Dr. Flint, in a lecture at Bellevue, thus summarizes the results of a number of cases :

From the study of these cases it may be conceded :

1. That by the application of cold water externally in cases of typhoid fever, the temperature of the body may, after a variable time of the continuance of the employment, be reduced to 102° or lower.

2. After a period varying very much in different cases, and, also, at different times in the same case, the temperature, as a rule, again rises as high as, or higher than, before the reduction.

3. Repeating the employment of cold as often as the axillary temperature exceeds 103°, the number of repetitions required in different cases is extremely variable.

4. The sponge bath and the wet sheet with sprinkling may be employed to the exclusion of the bath-tub in the antipyretic treatment in cases of typhoid fever as well as of other febrile diseases.

5. These modes of employing cold water may be continued sufficiently long for the reduction of temperature to 102° or lower, and repeated as often as may be required, without risk of any immediate injury, and the study of these cases furnishes no ground for supposing that a liability to complications or accidents is thereby increased.

6. Reduction of temperature by these modes as often as it rises, in the axilla, above 103°, improves the condition of the patient. The cases now studied do not afford proof, either that the fatality of typhoid fever, or that its duration is thereby diminished. The study of these cases, however, renders it possible that this proof would be afforded by a larger collection of cases.

During the period that the cases now studied were treated, seven hospital cases were recorded in which antipyretic treatment was not employed. In most of these cases the temperature did not rise above 103°, and it was for this reason that the treatment was not employed. Of these seven cases three were fatal, but I need not say that it would be unfair to draw any deduction from the contrast as regards the proportionate number of fatal cases. It is well known that, in general, resistance, toleration, and recuperation are not as well exemplified within as outside of hospitals. Moreover, in cases of typhoid fever, patients are not admitted into hospitals until some days after the commencement of the disease. The clinical test of therapeutical measures, as far as fatality is concerned, is therefore best afforded by the study of cases in private practice.

7. The results of the analysis of these cases, although not sustaining the statements of Liebermeister and others respecting the controlling influence of the employment of cold externally in cases of typhoid fever, yet not only show this method of antipyretic treatment to be safe, but afford encouragement to employ it with the expectation of diminishing the severity of the disease and its danger to life.

**Cough and Phthisis.**—Prof. Bartholomew (Med. News and Abstract) says: I come now to another symptom—cough—which usually taxes severely the resources of the physician. As cough prevents sleep, destroys rest, and is exhausting, the patients are clamorous for relief. Much coughing is a peculiarly severe form of exercise, and demands suppression or amelioration for this reason. The expedients are almost past computation—a sure indication both of failure and intractability. The reflex irritation proceeding from the fauces may be allayed by a gargle of bromide of potassium, by brushing over the mucous membrane a one per cent. solution of carbolic acid, or by atomizing a solution of morphia. The combination of diluted hydro-bromic acid, and spirit of chloroform proposed by Dr. Fothergill, does very well sometimes, but in general is disappointing. In fact, there are no efficient substitutes for opium, and we must turn to this when the cough is severe and persistent. The most generally useful of the preparations and derivatives of opium is codeia. The grounds of its utility in cough are these: it has a selective action on the pneumogastric nerve, allaying irritability of its end organs; it is calmative and hypnotic, and as compared to morphia is less excitant and less nauseant. Codeia is adapted to those cases in which the cough is largely nervous. It may be combined with strychnia when there is vomiting, and with atropia or picro-toxine when the sweats are profuse. The following are examples: ℞. Codeiæ sulph., gr. x.; ext. hyoscyami, ℈j. M. ft. pil. no. xx. Sig. One pil every four hours. ℞. Codeiæ sulph , gr. xvj., strychniæ sulph., gr. j; atropiæ sulph, gr. $\frac{1}{8}$; acid sulph. dil. ʒ ij; aquæ ʒ vj. M. Sig. Ten to fifteen drops three times a day. Morphia may be substituted for codeiæ in any of these prescriptions, by reducing the quantity one-half. Carbolic acid exercises no little influence over cough, expectoration, and fever, and is most serviceable in allaying the reflex vomiting. It is best given in solution, as follows: ℞. Acid. carbolic; gr. viij, aquæ laurocerasi, aquæ, aa ℥ i. M. Sig. A teaspoonful every four hours. Carbolic acid is especially indicated in the fetid expectoration of bronchiectasis. In the cough of fibroid lung, and of the stage of deposit before softening in caseous pneumonia, I have had excellent results from the administration of iodide of ammonium in the wine of tar. ℞. Ammonii iodid. ʒ ij; vini, picis liquid, syrp, tolu aa ℥ ij. M. Sig. A teaspoonful.

**Night Sweats.**—This is both an interesting and an important subject. It is interesting because of the striking results obtained by some new contributions to our therapeutical resources, and important because of the baleful influence of the sweats over the progress of the disease. If the sweats are profuse, there is an actual loss of material, of salts and organic matter, which represents waste of tissue. It is highly important to check this waste and preserve the material for the nutrition of the body. Until the recent observations of Dr. Murrell proving the great value of picrotoxine, atropia had the first place as a remedy for the night sweats of phthisis. I have usually given atropia with strychnia and morphia for the triple object of arresting the sweating, allaying cough, and stopping the reflex vomiting. It is not a little remarkable that the temperature is generally reduced by the combined use of these remedies. I have preferred to give the atropia with regularity three times a day, because of an influence over the progress of the disease, which seems to be independent of its anhydrotic power, and which can be explained only on the supposition that it has the power to improve the nutrition of the lungs. Its introduction into use as a remedy for phthisis has put a new phase on the prognosis of cases of caseous pheumonia, not advanced to the stage of softening. Some practitioners prefer to give atropia in a single full dose (1.60th of a grain) at bed hour, but the results are better, if given in a small quantity (1.200th of a grain) the effect being distributed through the day. The susceptibility to the action of atropia varies greatly, and as immense discomfort may be produced by a medicinal dose, care is necessary to avoid unpleasant results. Recognizing the importance of the influence which atropia appears to have on the trophic system of the lungs, I have had patients take it for years at a time, and without any ill effects.

The success of picrotoxine as an anhydrotic has been quite decided. Dr. Murrell finds that so small a quantity as the 1.200th of a grain, at bed hour, may arrest the sweating for a number of days. Although possessed of properties somewhat like those of strychnia, it is by no means so powerful, and may be given by the stomach up to 1.30th of a grain. Dover's powder, and, oddly enough, pilocarpine occasionally, acts very decidedly as an arrester of perspiration—a capital illustration of certain kinds of physiological antagonism. Take it all in all, atropia should be preferred in most cases, because of its apparent influence over the nutrition of the lungs. Whilst atropia is given, three times a day, picrotoxine may be exhibited at bedtime, if the effects of the former is inadequate.

**Does Vaccination Protect?**—It is becoming somewhat tiresome to have to reiterate the truths about vaccination. And it will sometimes occur to us that the best way, after all, would be to leave the question alone, and let the people find out the facts for themselves. Why should the doctor worry himself? If the people do not and will not believe in vaccination, why not drop our quills, and, having vaccinated ourselves and families, let the disease work away in its old-fashioned seventeenth century style. In 1821, half of the city of Boston lay sick with the small-pox. Would not the return of such a visitation be better than any pamphlets or statistics?

We present here a very few of the facts and figures upon which this universal agreement is based. It should be remembered, however, that aside from statistical proof, nearly every physician, at some time during his life, gets personal evidence of the efficacy of Jenner's discovery.

Previous to the introduction of vaccination, the annual mortality from small-pox throughout Europe was about three thousand to every million inhabitants. During the forty years subsequent, the mortality from the same cause was reduced in Sweden to 158 per million; in Westphalia to 114; in Bohemia, Moravia, and Austrian Silesia, to about 200; in Copenhagen to 286; in Berlin to 176; in England to 200.

In 1853, the Epidemiological Society received two thousand letters from medical men, affirming their belief, from personal experience, in the protective power of vaccination. Dr. Simon obtained similar answers from five hundred and forty-two to whom letters were addressed.

One observer, Marshall, has shown that among 757 persons exposed to small-pox, 231 had been vancinated, and of these latter only 24 took the disease. All the remainder, except seven, were infected. In 1871, an anti-vaccination excitement was fomented in England, and Parliament undertook to investigate the question. A large committee was appointed, and testimony taken from every quarter. The result was an overwhelming refutation of the points claimed by the anti-vaccinationists.

In the Franco-Prusian war, an epidemic of small-pox arose among the unvaccinated inhabitants of Brittany. It spread among the French soldiers, and destroyed 23,000 of them. The Prussian army, which was frequently and extensively exposed, and was larger than the French, lost only about 250 persons by the disease. The Prussian army was thoroughly vaccinated, the French was not. In the whole Prussian army for twenty years, though often exposed, only four fatal cases of small-pox occurred among the revaccinated.

There are a few things which have to be borne in mind when arguing for vaccination:

1. Vaccination is protective only when the virus is good, and has entered and affected the system.

2. There is a very small number of persons who will take small-pox if exposed, whether they have been vaccinated or not. If vaccinated, however, most of these will have the disease less severely.

3. Vaccination protects in most cases only for a certain period of years, and revaccination is necessary.

4. Vaccine lymph may possibly deteriorate after passing through the human system many times. This deterioration of humanized lymph has probably taken place in England, and perhaps elsewhere in Europe.

5. Small-pox varies in malignancy with the epidemic. There is no evidence, however, to prove that small-pox is any less malignant now, on the whole, than it was eighty years ago.

The above facts have to be considered in passing judgment upon the so-called statistics of anti-vaccinationists.—[Medical Record.

**J. J. Connor, M. D.,** of Palmer, Ill., reports a case in the Medical and Surgical Reporter, where a woman, being taken with an epileptic fit, fell into a shallow creek and was drowned.

The woman was about eight months and twenty days gone in pregnancy. As near as could be determined, the woman fell into the creek before eleven o'elock, A. M., and when seen by the doctor a few minutes after twelve M., was cold and dead; the fœtal heart-beat could at this time, be plainly heard and the movements of the child distinctly felt, and at 1:30 P. M., when the doctor left, the heart-beat and movements were as appreciable as when he first saw her, and the woman who laid the deceased out, "said they felt the fœtal movements up to near 2 P. M."

This case is very interesting as it is generally believed, by the profession, that the fœtus must die very shortly after the mother's heart ceases to beat, yet from this case it appears that there are instances where it may live for a considerable length of time. The doctor's excuse for not delivering the child at once, on discovering that it was alive, that the principal men of the village and an irregular practitioner of medicine said "that it would never do in the world, and that the child would not live anyway"—is a very lame one.—[N. W. Lancet.

**The Immediate Arrest of Bleeding from the Nose.**—John Kent Spender, M. D., in British Medical Journal, says: An improved instrument is described in Mr. W. Spencer Watson's book on Diseases of the Nose and its Accessory Cavities. It "consists of a gum elastic tube about five inches long, with lateral perforations near the end, and covered with thin caoutchouc membrane in the form of a spirally twisted bag for the last three or four inches of its length. To use it the membranous bag is smoothly folded over the continued tube, and the whole being oiled (diluted glycerine is better) is passed along the floor of the nares till it reaches the pharynx. The bag is now inflated, . . . and if a stop-cock is fitted the air is kept in by turning it as soon as sufficient tension is obtained." The cavity of the twisted bag could be injected with water if it were desired, but I have never found this necessary. When I recolleet what "bleeding from the nose" was in old days, I cannot be too thankful to Dr. Rose for his simple and effective invention. To be called to an obstinate accident of this kind, especially when other medical men had failed, was enough to make one sick at heart from the possibility of adding another failure to the dreary history; and then there was the consciousness that delay might mean impaired health or even death to the victim. The victory is half won when a man is armed with an apparatus which he knows is sure to succeed; and I am now speaking of cases in which he wishes to succeed, and which are not forms of natural blood-letting to be encouraged. The object of this brief communication is to recommend Dr. Rose's instrument for (1) facility of introduction; (2) the extent and evenness of the inflated area; and (3) the possibility of its remaining in situ for thirty-six or forty-eight hours, when it may be gently removed, and the hemorrhagic nostril can be syringed with some cold astringent fluid for purposes of cleanliness and the washing away of the blood debris.—[Chicago Medical Journal.

**Expressing the Placenta.**—The method at present in vogue of expressing the placenta is associated indissolubly with the name of Crede, for though the value of friction, of kneading, and compression was appreciated, as their writings show, by Mauriceau, Robert Wallace Johnson, Joseph Clarke, Busch, Mayer, and others, it remained for Crede to elevate placental expression to the rank of a recognized procedure of obstetric practice.

Crede's method consists essentially in applying at first light and afterward stronger friction to the fundus of the uterus till an energetic contraction is obtained; at its height the uterus is grasped so that the fundus rests in the palm of the hand with the fingers to the front. The exercise of circular compression forces the placenta from the uterus, or in case of failure the process may be repeated until the object is accomplished. It is true that the expulsion of the placenta will, as a rule, occur spontaneously. The unaided uterus is, however, liable to relax and become the source of hemorrhage; or where the delivery does not take place speedily, it may on the other hand close down so as to imprison the placenta within its cavity. The great merit of Crede's method is that by maintaining retraction it prevents hemorrhage, and by promoting speedy expulsion it guards against the dangers of retention When systematically practiced the bugbear known as adherent placenta is the rarest of accidents.

The practice is not difficult and is devoid of danger. To be successful, however, expression should be practiced only during a contraction, and the propulsive force should be directed from the fundus downward in the axis of the uterus. Spiegelburg lays great stress on exercising compression of the uterus from the moment the head emerges from the vulva, and not waiting until the delivery of the child is ended. By so doing general contractions are maintained and the detachment of the placenta promoted.—Lusk's new work on Midwifery.—[Medical Weekly.

**Cutaneous Eruptions Caused by the Use of Certain Medicines.**—(Giron. It. del' Malatt. Vener e del' Pelle, June, 1881).—Auspitz, in his valuable "System der Hautkrankeiten," gives the following as the secondary result of an angioneurasis:

QUININE.—(*a*) Scarlatinous erythema; (*b*) morbillious papular erythema; (*c*) hemorrhagia and purpura; (*d*) wheals, œdema, pruritus.

CINCHONA, BELLADONNA, STRYCHNINE, AND STRAMONIUM.—Manifestations like papulæ sudorales.

DIIGTALIS.—Erythema after a few days' use.

ACONITE.—Vescular exanthema.

SANTONINE.—Vesicles, wheals.

RHUS VENENATA AND TOXICODENDRON.—Vesicular eruption.

OPIUM AND MORPHINE.—Erythema, papular eruption with much desquamation and prrritus.

PILOCARPINE AND ATROPIA(?)—Augmentation of the perspiration.

PHOSPHORUS.—Purpura.

PHOSPHORIC ACID.—Bullous eruption.

MERCURY (internally).—Erythema, eczema.

ARSENIC.—Erythema and papules, eczema.

CARBOLIC ACID.—Erythema, vesicles or wheals.

SALICYLIC ACID.—Purpura, vesicles with laryngeal catarrh; wheals.

CHLORAL HYDRATE.—Erythema (well-colored), pruritus, desquamation; purpura and petechiæ; eczema with crust and scab.

BALSAM COPAIBA, CUBEBS, TURPENTINE.—Vesicles, erythema, eczema.

COD-LIVER OIL.—Acne.

IODIDE OF POTASH.—Papules; vesicles and bullæ; pustules and ecthyma; eczema; echymosis and purpura.

BROMIDE OF POTASSIUM.—Papules and pustules; deep tubercles and ecchymosis; vesicles; ulcers.—[Virginia Med. Monthly.

**Hypodermic Injection of Water in the Treatment of Pain.**—In the Gacete Medical of Venezuela, Dr. Ponte relates his experiences in several instances in which he employed water hypodermically for the relief of pain. The first case was that of a boy who was suffering from an intercostal neuralgia, so severe as to almost endanger the life of the patient by interference with respiration. Not having any morphine with him, the author determined to work upon the imagination of the sufferer by injecting pure cold water over the location of the pain, a procedure which, much to his astonishment, was followed by permanent relief. Impressed with this fact, Dr. Ponte resolved upon further experiments. The next case was one of toothache. In order to eliminate the imaginative element, he informed the patient of the treatment to be employed, for the execution of which permission was rather reluctantly given. An injection practiced upon the side of the face nearest to the pain, was followed by considerable ardor, but in less than a minute the odontalgia had subsided. Animated with these results, he employed cold water injections in a variety of different pains, always with happy issue, even in cases where morphine had been the drug previously administered. Another patient had been suffering nine years from intense gastro-intestinal neuralgia, which baffled all remedies. The pain came on after meals, and its violence was such as to cause her frequently to faint. When first seen by the writer, she was utterly prostrated. Two injections relieved the pain, and subsequent tonic treatment restored her to health. Several hundred cases have been treated in the manner described, with good results. No explanation is given as to the action of the remedy.—[New York Medical Record.

**The Specific Germ of Gonorrhœal Pus.**—After many unsuccessful attempts, a specific microbe has been discovered in the pus of gonorrhœa. The Annales de Dermatologie has a synopsis of a recent work by M. Weiss. The pus examined came both from men and women, and was taken with all the necessary precautions. In every case microscopic examination showed in pus corpuscles and epithelial cells little bodies, in some cases isolated and in others united in groups, and arrayed in a peculiar manner.

These bodies, of which the author gives a minute description, have always a characteristic appearance. M. Weiss examined the pus from thirty-two patients, and each time he found the parasitic forms. As a control experiment he examined the pus from cases of non-specific urethritis, balanoprostatites, bubo, leucorrhœa, etc., and never could discover the elements which he looks upon as characteristic of gonorrhœa. There still remain to be made culture experiments, which have not yet been begun.

M. Weiss calls especial attention to the action of hypermanganate of potassium on the parasite. In all cases of vaginal gonorrhœa treated in the service of M. Spillman, of Nancy, by means of injection of this salt, the parasites diminished rapidly in number, their enveloping zone disappeared and changes in appearance took place which showed either their destruction or at least great alteration as a result of the application of the salt. The strength used was 0.25 centigrammes to 1000.—[Jour. de Med. et de Chir. pratiques, November, 1881.—Exchange.

**Hypodermic Injection of Quinine.**—For injection the following solution was made: Thirty grains of sulphate of quinine, fifteen grains of tartaric acid, and half an ounce of water. Twenty minims of this were injected every two hours in a case of intermittent fever. The patient had been a resident for some years in the West Indies, and when there several times had ague. He stated that he knew when it was going to recur since he returned to this country, and that he had been ordered to take quinine. This, however, always occasioned vomiting, and he was obliged to desist and endure the fever, which lasted forty-eight hours. The injection which was given caused no pain, and it was followed by no abscesses. It seemed to have the effect of warding off the attack, according to the man's statement. On several occasions this method of administering quinine has been tried. I can testify as to the pain being nil, and to the absence of any inflammation. Dissolved in any other acid quinine is a painful and troublesome agent, and its use cannot be recommended. In cases of typhoid fever I would not hesitate to use it hypodermically, and should imagine that good results would ensue, both in reducing the temperature and in preventing any derangement of the digestive organs, apt to be induced by large doses administered by the mouth.—[Therapeutic Gazette.

**On the Indications for the Use of Morphia in Pureperal Eclampsia.**—Dr. McFarlane, of Toronto, who has found morphia, administered hypodermically, to be a most efficient remedy in the treatment of puerperal convulsions, gives, in the Canadian Journal of Medical Sciences, for December, 1881, his reasons for regarding it as especially indicated in this disease. He first points out what he regards as the true cause of the convulsions, viz., anæmia of the brain, with increased irratability and general exhaustion of the entire nervous system, resulting from the disturbance of the circulation produced by the increased labor required of the heart in carrying on the fœtal circulation. In morphia we have a drug which produces an increased flow of blood to the nerve centres, and by its soporific effect allows the brain to rest while increased power is gained to carry on the nervous functions of the body. The control which morphine exercises over the disease, both in the preliminary stage as well as when the convulsions actually set in, is so prompt and decisive, he says, as to convince the most skeptical after having given it a fair trial. To give any preparation of opium in this disease by the stomach, he regards as of little if any use, as the sickly condition of the organ is such that the medicine is not absorbed in time to be of any benefit to the patient, and moreover, the remedy should be given early and in sufficient quantity (not less than from one-half to a grain) to control the convulsions at once.—[The Medical and Surgical Reporter.

**Manipulation of the Scapula in Dislocation of the Shoulder.**—The patient being completely stripped as far as the upper part of the body is concerned, is either made to lie on a couch or a bed, or he can be, from my last experience, easily manipulated in a sitting posture. Take for example dislocation of the left shoulder. The left wrist is grasped with the left hand, and the arm gently abducted; the fingers of the right hand are then firmly pushed between the head of the humerus and the wall of thorax, when with a sweep of the arm across the body the head of the bone is easily lifted and slides into the glenoid.—[E. T. T., in British Medical Journal.

**Calomel and Chlorate of Potash.**—A correspondent of the Medical and Surgical Reporter relates the following to prove the incompatibility of the two compounds:

C. W., aged four years, suffering from an attack of pharyngitis (acute). I ordered a purgative dose of calomel, and at the same time a prescription containing tinctura ferri chloridi and potass. chlorat., the latter not to be given till the purgative effects of the former had been obtained; but contrary to my directions, in about an hour after giving the calomel they gave a dose of the iron and potash.

A short time afterwards I was summoned, and found my little patient suffering from all the symptoms of poisoning by corrosive sublimate, but with no salivation, nor did any appear subsequently. I administered the usual remedies for such poisoning, and the recovery was rapid.

[The addition of the tincture of iron entirely changes the aspect of the question. The tincture is always decidedly acid, sometimes quite strongly so. The presence of a powerful mineral acid like muriatic acid will cause chemical decompositions which would not occur otherwise.]—[Druggists' Circular.

**Rattlesnake Poison.**—Dr. L. Filho has published the following results of his experiments on the poison of the rattlesnake in the Archivos do Museu Nacional do Rio de Janeiro: 1. The poison of Crotalus horridus acts upon the blood by destroying the red blood-corpuscles, and by changing the physical and chemical quality of the plasma; 2. The poison contains some mobile bodies similar to the micrococcus of putrefaction; 3. The blood of an animal killed by a snake's bite when inoculated to another animal of the same size and species causes death of the latter within a few hours, under the same symptoms and the same changes of the blood; 4. The poison can be dried and preserved for a long time without losing its specific quality; 5. Alcohol is the best antidote to the poison of Crotalus horridus known at present.—[Louisville Medical News.

**Dr. R. Fowler, of Ednaville, Texas,** writes in Southern Clinic as follows: A case of a little two-and-a-half year old child was to-day presented for treatment. The case is unique; it consists of a small worm under the skin, crawling around the body; and as proof of the truth of it, in October last Dr. Emanuel took one out of the same child. The father says it was of a white color, with a flat, black head, and about two-and-a-half inches long. It comes near the surface at times, and then recedes away. You can readily see the track beneath the skin by the ecchymosed appearance. It was on the shoulder yesterday morning. I could easily destroy it by hypodermic injection, but I wish to get it out entire, and send to some entomologist for identification. Do you know of any such case on record? And what entomologist or microscopist must I send it to? The child is perfectly healthy every way; is fat, and it gives him no pain or trouble. But how came this worm there? How did it get in the cellular tissues?

